# Early Ovariectomy Results in Reduced Numbers of CD11c+/CD11b+ Spleen Cells and Impacts Disease Expression in Murine Lupus

**DOI:** 10.3389/fimmu.2016.00031

**Published:** 2016-02-15

**Authors:** Melissa A. Cunningham, Jena R. Wirth, Jennifer L. Scott, Jackie Eudaly, Erin L. Collins, Gary S. Gilkeson

**Affiliations:** ^1^Division of Rheumatology and Immunology, Ralph H. Johnson Veterans Affairs Hospital, Medical University of South Carolina, Charleston, SC, USA

**Keywords:** systemic lupus erythematosus, mouse models, estrogen, ovariectomy, dendritic cells

## Abstract

Ninety percent of those diagnosed with systemic lupus erythematosus are female, with peak incidence between the ages of 15 and 45, when women are most hormonally active. Despite significant research effort, the mechanisms underlying this sex bias remain unclear. We previously showed that a functional knockout of estrogen receptor alpha (ERα) resulted in significantly reduced renal disease and increased survival in murine lupus. Dendritic cell (DC) development, which requires both estrogen and ERα, is impacted, as is activation status and cytokine production. Since both estrogen and testosterone levels have immunomodulating effects, we presently studied the phenotype of NZM2410 lupus-prone mice following post- and prepubertal ovariectomy (OVX) ± estradiol (E2) replacement to determine the impact of hormonal status on disease expression and DC development in these mice. We observed a trend toward survival benefit in addition to decreased proteinuria and improved renal histology in the early OVX, but not late OVX- or E2-repleted WT mice. Interestingly, there was also a significant difference in splenic DC subsets by flow cytometry. Spleens from NZM mice OVX’d early had a significant decrease in proinflammatory CD11c+CD11b+ DCs (vs. unmanipulated WTs, late OVX- and E2-repleted mice). These early OVX’d animals also had a significant increase in tolerogenic CD11c+CD8a+ DCs vs. WT. These data join a growing body of evidence that supports a role for hormone modulation of DCs that likely impacts the penetrance and severity of autoimmune diseases, such as lupus.

## Introduction

Systemic lupus erythematosus (SLE) is the prototypic autoimmune disease characterized by production of autoantibodies and immune complex-mediated end-organ damage. Nine out of 10 patients diagnosed with lupus are female, thus biologic sex is important in disease development. The cause of the sex difference in SLE is likely multifactorial, including the sex chromosomes, sex hormones, and their receptors. Perhaps the strongest epidemiological data to support a role for hormone impact on disease is the fact that incidence of disease is highest during the reproductive years when women are most hormonally active (in contrast to pre-menarche and post-menopause, when lupus incidence is lower and the female:male ratio is less profound). The SELENA trial showed that use of oral contraceptive pills in SLE patients did not enhance flare rates; however, estrogen replacement therapy in post-menopausal women did result in a slight increase in mild lupus flares, indicating that changing/replacing estrogen levels in an estrogen-deficient setting can impact disease (1, 2).

In murine models of lupus, manipulation of sex hormones also has significant impact on disease expression. NZB/NZW mice and some of the derived NZM strains, including NZM2410, have significant female predominance of disease (3). Both genders of MRL/lpr mice develop severe lupus; however, there is a trend toward earlier and more severe disease in females (4). In classic experiments by Roubinian et al. and later by Tarkowski et al., manipulation of sex hormones and castration led to significant effects on disease expression (5–10). Specifically, castration of male NZB/NZW or MRL/lpr mice led to female-like disease and ovariectomy (OVX) with androgen replacement in female mice led to disease protection. Protection in male mice was limited to those in which gonadectomy was performed at 4 weeks of age or earlier (prepubertal). Interestingly, castration *after* onset of sexual maturity had little impact on disease. Administration of pharmacological doses of estrogen led to significant enhancement of disease in ovariectomized females, castrated males, and unmanipulated females (6). These experiments are suggestive that there is an imprinted component to female sex that is fixed at the time of sexual maturity. This imprinting appears manipulatable since lupus can be induced by repleting estrogen. Once the imprinting has occurred, however, taking away estrogen does not appear to be an effective therapeutic intervention, although there is modest data to support androgens as a treatment.

Estrogen has pleiotropic effects on many different cell types, including immune cells. For example, estrogen treatment of macrophages is proinflammatory and induces expression/release of TNFα and nitric oxide (NO) (11–16). In contrast, estrogen given to macrophage in the setting of LPS treatment results in estrogen partially inhibiting the release of NO and TNFα (15). This estrogen-mediated immune suppression was mediated *via* the activation of ERα. Short-term administration of exogenous estrogen appears immune suppressing, while long-term exogenous or endogenous estrogen is proinflammatory (17). In some lupus-prone strains, exogenous estradiol, *via* ERα, promotes survival of DNA-reactive B cells and decreases the activation threshold for BCR signaling, leading to increased autoantibody formation (18–20). While the ability of estrogen to impact B cell activation requires classic ERα action, estrogen effects on B cell survival do not (21). This may also explain data in NZM2410 ERαKO female mice that have high levels of both serum estradiol and anti-dsDNA but are still protected from renal disease and have a significant survival benefit (4). It seems clear that estrogen and ERα have differential effects on immune cells that may also be dose and time dependent.

In lupus, dendritic cells (DCs) play a critical role in disease pathogenesis and progression. For example, mice deficient in DCs (conventional DCs and most plasmacytoid DCs) are protected from developing lupus nephritis. DC-deficient mice have similar autoantibody levels and immune complex deposition as their WT littermates, but lack progression of renal damage despite ongoing immune complex deposition. As noted above, we previously showed that female NZM2410 ERαKO mice also had significantly less renal disease and prolonged survival compared to WT littermates despite similar serum levels of autoantibodies and glomerular immune complex deposition. We subsequently showed that DCs from ERαKO mice have a blunted inflammatory response (e.g., IL-6, IL-1β, and IFNα production) to toll-like receptor (TLR) ligands (22). These findings suggested that an important effect of estrogen and ERα in lupus may be on the innate immune system and local tissue response to inflammation.

Despite significant research effort, the mechanisms underlying estrogen and its receptors effects on disease remain unclear. Since estrogen and ERα are known to modulate DC development *in vitro* (23–26), we and others are interested in the impact of estrogen on DCs in SLE, especially since estrogen influences DC development and function primarily *via* ERα (24, 26). Due to the importance of DCs in SLE pathogenesis, we investigated the effect of early and late OVX ± estradiol (E2) repletion on both lupus disease phenotype and DC development in NZM2410 mice.

## Materials and Methods

### Mice

Mice were maintained at the Ralph H. Johnson VAMC Animal Facility (Charleston, SC, USA). Animal protocols followed the principles outlined in the Guide for the Care and Use of Laboratory Animals, and were approved by MUSC’s IACUC. The NZM2410 mouse strain was acquired from Jackson Laboratory (Bar Harbor, ME, USA) and was maintained on a 12-h light/dark cycle with access to food and water *ad libitum*. All experimental mice (*n* = 34) were female and littermates where possible. Mice were ovariectomized at 4 or 8 weeks of age. One group of mice OVX’d at 4 weeks of age subsequently received 0.1 mg, 90-day time release 17β estradiol pellet (Innovative Research, Sarasota, FL, USA, Catalog number: NE-121) that was implanted subdermally. Mice were sacrificed with isoflurane and cervical dislocation at 32 weeks of age or when they reached sacrifice requirements (>10% loss of weight, >500 mg urine protein as assessed by dipstick, or upon recommendation by the animal facility).

### Serum Anti-dsDNA, Serum Estradiol, and Serum Testosterone

Serum was collected throughout the experiment and at time of sacrifice. Serum anti-dsDNA was measured by ELISA assay, as previously described (4). Estradiol levels were assessed *via* ELISA (Calbiotech, San Diego, CA, USA), with an assay sensitivity of 3 pg/ml; precision: 3.1% (intra-assay), 9.9% (inter-assay). Testosterone serum levels were assessed by radioimmunoassay (RIA) at the University of Virginia Center for Research and Reproduction Ligand Assay and Analysis core.

### Urine Protein Excretion

Mice were housed in metabolic cages for 24 urine hour collection at 2-week intervals starting at 10 weeks of age until sacrifice. To prevent bacterial growth, antibiotics (ampicillin 25 μg/ml, gentamicin 50 μg/ml, and chloramphenicol 200 μg/ml) were added to the collection tube. After 24 h, urine quantity was determined and samples were frozen at −20 for future analysis *via* mouse albumin ELISA with known standards.

### Spleen and Kidney Processing and Renal Pathology

Spleens were harvested and kept on ice during processing. The spleens were processed through 40 μm strainers and depleted of red blood cells with red blood cell lysis buffer (144 mM NH_4_Cl and 17 mM Tris, pH 7.6). Spleen cells were washed twice with cold RPMI before being stained for flow cytometry analysis.

One kidney was divided evenly for renal pathology and immunofluorescence. One half was snap frozen in liquid nitrogen and stored at −80°C for immunofluorescence analysis, the other half was fixed with buffered formalin, embedded in paraffin, and then sectioned and stained with hematoxylin and eosin. Kidney sections were analyzed in a blinded fashion by Dr. Phillip Ruiz (Department of Pathology, University of Miami School of Medicine, Miami, FL, USA) and graded on glomerular hypercellularity, segmental mesangial expansion, neutrophils/cell debris, crescent formation, and necrosis. These grades were combined for a total glomerular and interstitial pathology score. Deposition of IgG and complement component C3 was analyzed on the half kidney snap frozen in liquid nitrogen. Deposition was assessed by immunofluorescence by incubating slides with rabbit anti-mouse IgG FITC (Sigma) and sheep anti-mouse C3 FITC (Sigma). IgG and C3 were graded 0–3 for intensity of staining, as previously described (4).

### Staining and Flow Cytometry

Spleen cells (4 × 10^6^ per sample) were isolated in staining buffer (0.5% BSA and 0.02% sodium azide in 1× PBS) and stained with CD11c-Brilliant violet 605 (1:100), CD8a-Brilliant violet 421 (1:100), and CD11b-PE (1:400). Cells were incubated with antibodies for 30 min on ice in the dark. All antibodies were purchased from Biolegend (San Diego, CA, USA). Viability was assessed using LIVE/DEAD Fixable Dead Cell stain (Life Technologies, Carlsbad, CA, USA) at a concentration of 50 μl/million cells. Cells were washed twice with staining buffer and resuspended in 0.3 ml of staining buffer for flow cytometry. Cells were acquired on an LSRFortessa cell analyzer (BD Biosciences, San Jose, CA, USA), and analysis was performed using FlowJo software (FlowJo LLC, Ashland, OR, USA).

### Statistical Analysis

Kruskal–Wallis one-way analysis of variance and *post hoc* Dunn’s multiple comparison test were utilized to test for significance when comparing treatment groups. SEMs were reported where applicable. *p* Values ≤0.05 were considered significant. Log rank analysis was used to compare trends in animal survival.

## Results

### Effects of Early vs. Late Ovariectomy on Survival, Body, and Organ Weights in NZM2410 Mice

**Figure 1:**
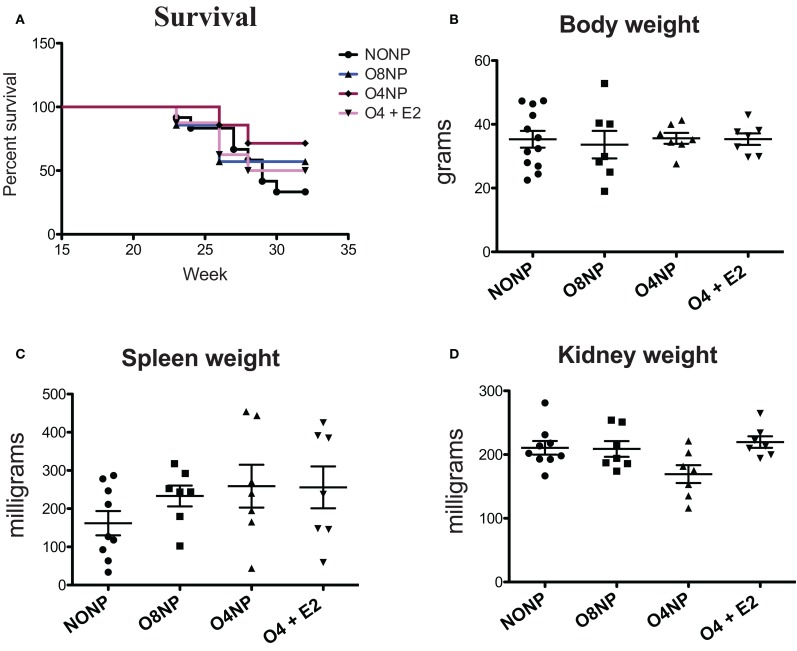
**Health parameters in NZM2410 mice**. NZM2410 lupus-prone mice were unmanipulated (NONP, *n* = 12), OVX’d after puberty (O8NP, *n* = 7), OVX’d before puberty (O4NP, *n* = 7), or OVX’d before puberty and administered estrogen (O4 + E2, *n* = 7). **(A,B)** No significant difference in survival or body weight between the four groups was observed, although there was a trend toward survival benefit in the O4NP group (71%) vs. WT NONP (33%). **(C)** No significant difference in spleen weights between NZM2410 WTs based on hormonal milieus was observed, although there was a trend toward bigger spleens in OVX’d mice. **(D)** No significant difference in kidney weights between-treatment groups, but a trend toward reduced kidney weight in O4NP vs. O4 + E2 was noted (*p* = 0.07).

NZM2410 mice underwent four different treatments: (1) no OVX and no E2 pellet (*NONP*), (2) late OVX at 8 weeks (post-puberty) with no E2 pellet (*O8NP*), (3) early OVX at 4 weeks (pre-puberty) with no E2 pellet (*O4NP*), or (4) early OVX at 4 weeks with 0.1 mg E2 *via* sustained release pellet (*O4* + *E2*). Similar to our previous work (4), unmanipulated NZM2410 (NONP) female mice began to die at 23 weeks of age and only 33% survived to 32 weeks (study endpoint). In contrast, as shown in Figure 1, 71% of mice OVX’d before puberty (O4NP) survived to 32 weeks of age. Mice that were either OVX’d late (post-puberty), or early but estradiol repleted (O4 + E2) were less protected, with only 42 and 50% surviving to 32 weeks of age, respectively. Overall, there was not a significant difference in survival between the four treatment groups (Figure 1A). At the time of sacrifice, all mice were weighed and total body, spleen, and kidney weights were documented. Neither OVX nor presence of E2 pellet significantly altered the weights of treated mice compared to the unmanipulated NONP mice (Figure 1B). Spleen weights were variable within all four treatment groups, but no significant differences were observed between groups (Figure 1C). Although O4NP mice trended toward lower kidney weights compared to the other groups (Figure 1D), there was again no significant difference observed. Overall, OVX with or without E2 repletion did not significantly alter disease penetrance in NZM2410 mice; however, early OVX did appear to impact disease and offer modest protection.

### Serum Anti-dsDNA Autoantibody and Proteinuria Levels in OVX’d NZM2410

**Figure 2:**
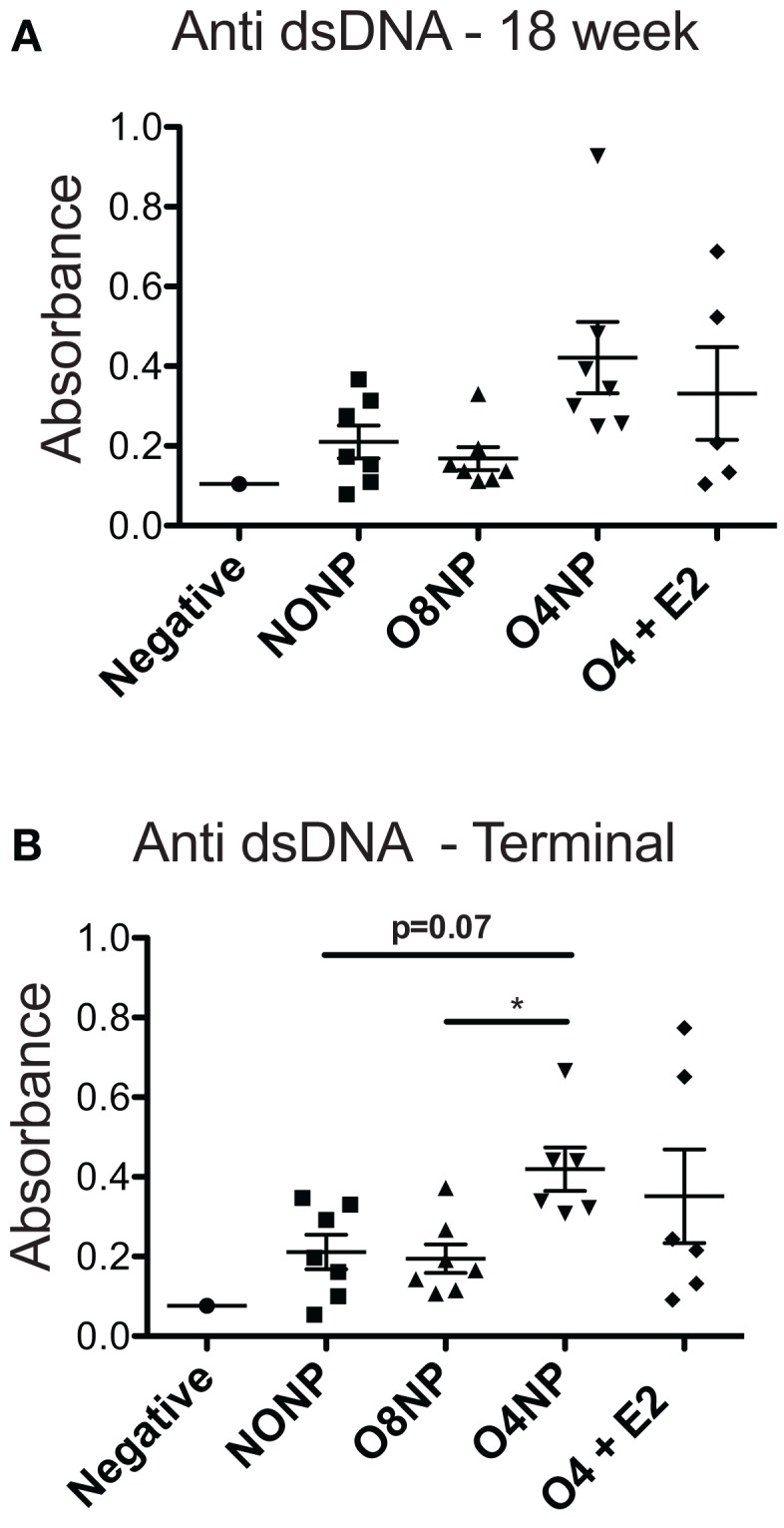
**Anti-double stranded DNA levels in NZM2410. (A)** 18-week anti-dsDNA levels did not show any significant differences among treatment groups. All NZM mouse groups had elevated levels compared to the negative control, a C57BL/6J mouse that produces no autoantibodies. **(B)** At the terminal endpoint, O4NP mice had significantly increased levels of dsDNA compared to O8NP. Elevated anti-dsDNA levels from O4NP mice also trended towards significance when compared to unmanipulated NZM (*p* = 0.07). All results shown as mean ± SE with statistical analysis done by Kruskal–Wallis non-parametric test with *post hoc* Dunn’s multiple comparison test, *p* < 0.05.

**Figure 3:**
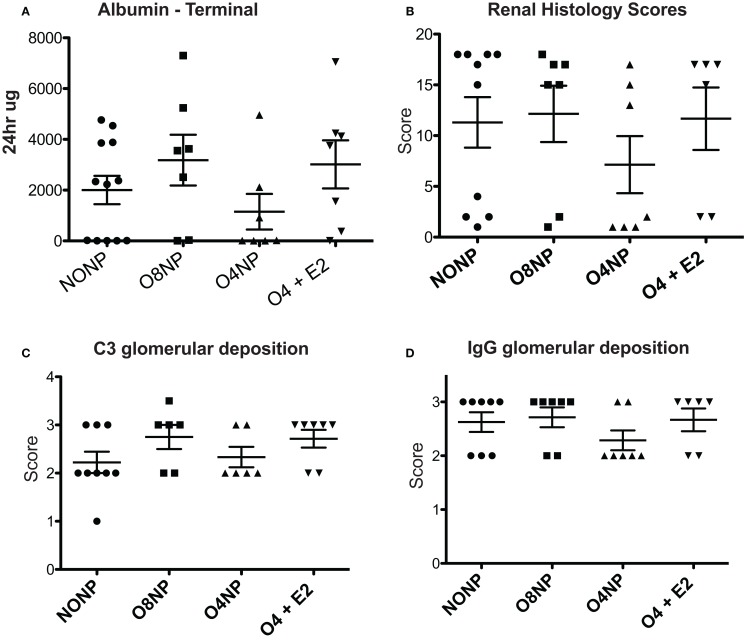
**Renal pathology in NZM2410 mice**. **(A)** Terminal albumin levels did not show significance across experimental groups, O4NP mice trended toward reduced levels. **(B,D)** C3 deposition was assessed *via* immunofluorescence after incubating slides with sheep anti-mouse C3 antibody. Slides were scored on a 0–3.5 scale. No significant difference was seen between groups. IgG deposition was assessed in a similar manner to C3 with slides being stained using rabbit anti-mouse IgG antibody. Again, no significant difference in treatment groups was observed. **(C)** Histology was scored based on a variety of parameters: glomerular hypercellularity, segmental mesangial expansion, neutrophils/cell debris, crescent formation, and necrosis. Though the O4NP mice trended toward lower histology scores, no significant difference was observed in the treatment groups. All results shown as mean ± SE using Kruskal–Wallis non-parametric test with *post hoc* Dunn’s multiple comparison test, *p* < 0.05.

Terminal anti-double stranded DNA (dsDNA) antibody levels were assessed *via* ELISA. O8NP mice had anti-dsDNA levels similar to unmanipulated NZM. Interestingly, we observed increased anti-dsDNA levels in both groups of early OVX’d mice, which was significantly different in the O4NP mice (vs. O8NP) (Figure 2B). Similar results were observed in anti-dsDNA levels at the 18-week time point (Figure 2A). At 18 weeks of age, when onset of lupus-like kidney disease typically becomes evident in NZM2410 mice, 08NP and O4 + E2 mice had not developed proteinuria by albumin ELISA; unexpectedly, several O4NP mice had early levels of proteinuria, similar to unmanipulated NZM mice at that time point (data not shown). Nephritis in these mice did not progress; in fact, proteinuria in the same three O4NP animals was slightly reduced at the terminal time point. In the other WT mice, terminal albuminuria levels increased over time as expected reflecting an increased number of animals developing lupus renal disease (Figure 3A). It was unexpected to find both higher levels of anti-dsDNA and earlier onset of proteinuria in the O4NP animals (which had the best overall survival), and suggested that early OVX resulted in a disease phenotype that is less severe/less progressive, rather than delayed in onset.

### E2 Effects on Renal Pathology in NZM2410 Mice

Kidneys were collected at time of sacrifice and sectioned. C3 and IgG deposition, markers of kidney involvement in lupus disease, were determined by C3 and IgG immunofluorescence (IF). IF intensity was semiquantitated using a 0–3 scale by a blinded scorer. For both C3 and IgG, all four treatment groups scored a 2 or greater, indicating moderate to high levels of immune complex deposition that was not significantly different between groups (Figures 3B,D). H&E stained kidney samples were prepared and renal pathology scores were determined by a blinded pathologist who used multiple standard parameters to assess disease involvement of glomeruli, tubules, interstitium, and vessels. In agreement with the improved survival and reduced levels of terminal proteinuria observed, O4NP mice appeared to have less severe kidney disease by renal pathology score compared to the other treatment groups, although this decrease was not statistically significant (Figure 3C). Overall, all four groups showed evidence of renal disease by proteinuria, deposition scores, and overall renal pathology scores, although O4NP mice were modestly protected.

### Hormone Levels in NZM2410 Mice with and without E2

**Figure 4:**
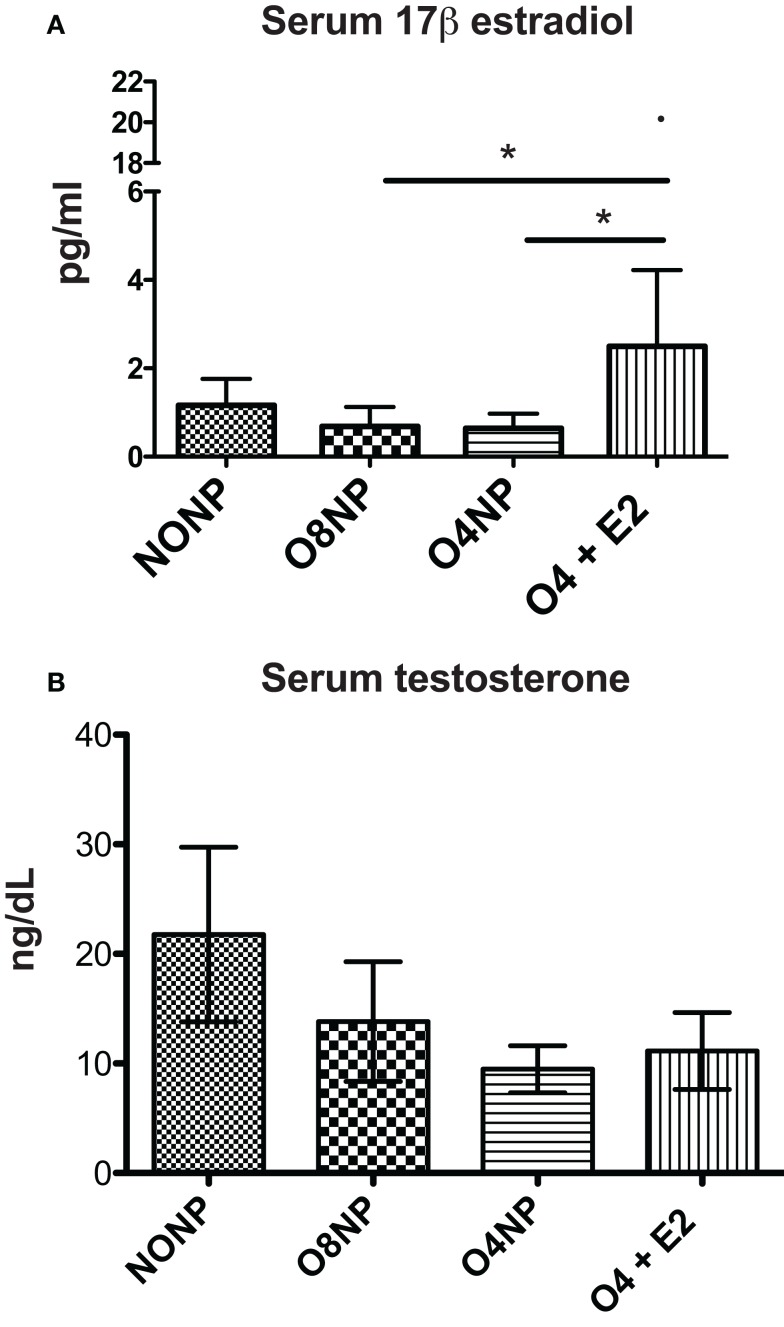
**Hormone levels in NZM2410 mice**. (A) serum 17β-estradiol levels in terminal serum samples were assessed *via* ELISA. O4 + E2 mice had physiological levels of estrogen after pellet placement with one outlier (20.78 pg/ml) having much higher documented levels. The O4 + E2 group had significantly higher levels compared to O4NP and O8NP animals, which did not have exposure to exogenous estrogen. **(B)** Serum testosterone levels in terminal serum samples were determined with radioimmunoassay with no significant difference being seen between treatment groups.

Estradiol levels in mice can range from <5 pg/ml (old, sick, or non-breeding) to >1000 pg/ml (young pregnant). In this study, serum 17β estradiol levels for all treatment groups were assessed by the current gold standard 17β-estradiol ELISA (27). The O4 + E2 treatment group was OVX’d and had estrogen replaced *via* subcutaneous E2 pellet to account for potential effects of varying endogenous estrogen levels in cycling NZM2410 mice. The E2 pellet in O4 + E2 maintained an average of 3.8 pg/ml estrogen during the experiment, significantly higher than estrogen levels in the OVX’d unrepleted treatment groups, as expected (Figure 4A). O4 + E2 mice maintained a level of physiological estrogen that is normally seen in mouse strains, with one individual having a much higher level of estrogen than the rest of the cohort. NONP mice had low normal estrogen levels which could be caused by a variety of factors including age, disease state, and hormonal cycle, since NZM2410 begin to manifest disease symptoms as early as 16 weeks of age. Terminal testosterone levels were quantified with RIA. NONP mice trended toward higher levels of testosterone, as would be expected in gonad-intact cycling female mice, compared to the three OVX’d treatment groups, but no significant differences were observed (Figure 4B).

### Flow Cytometry

**Figure 5:**
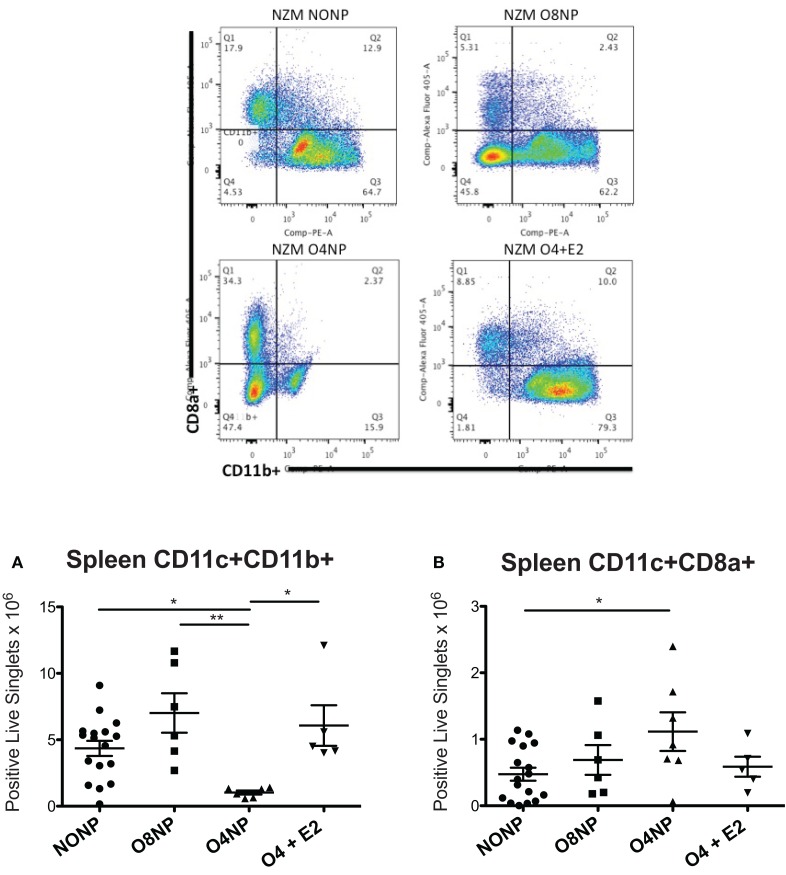
**Spleen DC flow cytometry in NZM2410 treatment mice**. Spleen cell populations were analyzed for absolute number of DCs. Gates were set on live singlets that were CD11c+, then subsequently gated on CD8a+ vs. CD11b+. Representative flow cytometry is shown in the four upper panels. **(A)** O4NP mice had a significant reduction in the number of cDCs observed by CD11c+/CD11b+ (vs. NONP, O8NP, and O4 + E2). **(B)** We also observed an increase in a tolerogenic DC subset (CD11c+/CD8a+) in spleens of O4NP mice vs. NONP.

Dendritic cells are dysregulated in SLE in both human disease and murine models of lupus. Since estrogen is required for normal DC development, we investigated the impact of post- vs. prepubertal OVX ± estrogen on DC numbers in lupus-prone NZM2410 mice. DC numbers were analyzed by flow cytometry using *ex vivo* splenocytes harvested at the time of sacrifice. We found that the O4NP mice, which tended toward a survival benefit, had a significant reduction in the number of CD11c+/CD11b+ cells (vs. NONP, O8NP, and O4 + E2) (Figure 5A). This was particularly surprising in the case of the profound difference between O4NP and O8NP, where the only difference between groups was the timing of the OVX, suggesting that hormone levels *during* pubescence are critical to determining the developmental program of hematopoietic progenitors. This concept is further supported by the *increase* in a different myeloid subset (CD11c+/CD8a+) from spleens of O4NP mice vs. NONP (Figure 5B). Importantly, CD11c+/CD8a+ DCs have been associated with promotion of regulatory T cells and mediation of tolerance in multiple tissue types and may partially explain the trend toward disease protection in the O4NP mice.

## Discussion

Sex biases in autoimmunity have been observed in both humans and in rodent models of disease (28). SLE is a disease with a profound sex bias. Although there are data to support a role for sex chromosomes, sex hormones, and their receptors in disease expression, a clear etiology for this health disparity is not apparent. It is known that both estrogens and androgens can modulate the expression of autoimmunity. As in human disease, the striking sex difference in most murine lupus strains includes females developing earlier, more severe disease. This can be modified in both sexes by castration and differential hormone replacement, and the timing of this appears to affect disease differentially in males (10, 29–31). While there is somewhat conflicting data from prior studies of OVX, estrogen replacement, and estrogen receptor knockout on autoimmunity and immune functions in lupus animals, there is more agreement on the generally protective role for androgens in disease expression. In seminal work by Roubinian et al., prepubertal castration of male NZB/NZW F1 (B/W) lupus-prone mice resulted in accelerated disease development, with both accelerated autoantibody production and mortality. In contrast, prepubertal castration of female mice did not change mortality, although it did have minor effects on autoantibody production and class switching (10). The effect on castrated males and females was much greater when they were treated with androgen vs. estrogen therapy, which resulted in attenuated kidney disease and improved survival when castrated females were treated with androgen, while castrated male B/W mice treated with estrogen had accelerated disease severity similar to that of WT females (29). Thus, androgen was protective while estrogen promoted disease. Anti-estrogen treatment with tamoxifen has also been shown to reduce autoantibody production and mortality in B/W mice (32, 33). These studies were some of the first to show evidence that androgens, estrogens, and anti-estrogens can modulate autoimmunity and disease expression in lupus.

The purpose of the current study was to determine the impact of early (prepubertal) vs. late (postpubertal) OVX on the disease phenotype and effects on DC development/subsets in female lupus-prone mice. We utilized NZM2410 mice, a recombinant inbred strain of New Zealand Mixed mice that have a highly penetrant disease with early onset. We have been using these mice for many years to study the effects of estrogen receptor alpha (ERα) on disease expression. We previously showed that backcrossing ERαKO mice onto an NZM2410 background resulted in reduced kidney disease and significantly prolonged survival in female, but not male mice (4). Of note, ERβKO NZM mice were not similarly protected. Thus, it has not been shown that deficiency of ERβ, or the early lack of estrogen alone (such as in castrated female B/W mice), significantly impacts disease, although we and others have shown that deficiency of a functional ERα does impact disease (4, 34). Since the *timing* of castration in males leads to variable protection in some lupus strains, we undertook this study in WT NZM female mice to ovariectomize either before or after puberty (±estrogen replacement in the early OVX group). The results are consistent with earlier experiments that showed no significant impact on the development of lupus nephritis with OVX alone. Differences were noted, however, in the fact that estrogen replacement with a 17β-estradiol pellet, which resulted in physiological doses, did not exacerbate disease over WT animals. Additionally, anti-dsDNA levels were not different between the estrogen-treated group and the other groups, but surprisingly, were significantly different between the early and late OVX groups. This result supports the concept that there exists a time point during the transition between adolescence and adulthood, as in males, in which hormone levels critically impact development of autoimmunity. It is important to separate autoantibody production from disease; however, since the animals with higher anti-dsDNA levels trended toward reduced kidney disease and improved survival. This is similar to what we observed in our previous study of NZM ERαKO mice (higher anti-dsDNA, lower disease scores), lending credence to the concept of a two-step process, whereby autoantibodies are necessary but not sufficient for disease (a concept also supported epidemiologically by the large number of normal human females that have autoantibodies but no evidence of disease).

Since most testosterone in females is secreted by the ovaries, it is also important to mention that OVX’d mice have reduced levels of both testosterone and estrogen, as evidenced by serum levels in our OVX’d animals. One limitation of this study is that testosterone and estrogen levels were examined late: at 18 weeks (data not shown) and at the terminal endpoint. We chose these time points to reflect hormone levels during early and late lupus disease onset and to show evidence of successful (long-lasting) estradiol replacement. However, the drawback is that these levels do not reflect hormone levels during puberty onset, at which point testosterone and estrogen levels would have been different between the castrated mice (early OVX) and the gonad-intact (late OVX) mice. Also, testosterone levels were assessed by RIA, which is relatively sensitive and specific, but estrogen levels (using ELISA) are notoriously difficult to measure with accuracy. We did see lower estrogen levels in our unmanipulated animals than we expected, likely due to increasing disease activity and abnormal ovarian cycling at that late time point (these animals do not breed well beyond 20 weeks).

Importantly, estrogen has different effects depending on timing, concentration, and cell type. For example, estrogen can help break tolerance by promoting survival of autoreactive B cells and leading to increased autoantibody production in a murine lupus model (19, 35). In other settings, such as experimental autoimmune encephalitis (EAE), which is thought to be more T-cell driven, estrogen is neuroprotective/anti-inflammatory *via* ERα signaling in astrocytes, which are CNS APCs expressing MHCII, B7, and CD40, and are critical for T cell activation (36, 37). It is also likely that estrogen impacts immune cell types differentially depending on the milieu.

Dendritic cells are immune cells that are important for both maintenance of tolerance and an appropriate innate immune response. As with most immune cells, the development status of DCs is critical in influencing its cellular functions. Immature DCs mostly induce tolerance (since they express fewer surface molecules that give a second stimulatory signal), whereas mature DCs function to induce immune responses by stimulating proliferation of naïve antigen-specific CD4+ T cells or CD8+ T cells (38, 39). It is well known that DCs in SLE are abnormal in both number and function (40–42). Additionally, previously published studies by our lab and others revealed a critical role for estradiol and ERα modulating DC number and function in normal and lupus-prone animals (22, 25, 26, 43). Estrogen also influences maturation and function of human monocyte-derived DCs (44). One limitation to this study is that, although we focused on DCs, it is likely that our isolated cells, whether spleen derived or BM derived, did have some frequency of monocytes and macrophage present. We and others are moving toward better markers to gate or sort more specifically on DC subsets, but in this experiment, our markers are inadequate to identify DCs only.

Because dysregulated DC activation and function are implicated in the pathophysiology of SLE, and estrogen plays a role in SLE pathogenesis, this line of study is vital to our understanding of sex bias in this disease. In the current study, we observed that the single act of ovariectomizing female lupus-prone mice prior to puberty resulted in near depletion of absolute numbers of CD11c+/CD11b+ DCs from the spleen. This effect appears to be due to a lack of estrogen at a critical time point, since replacement of estradiol by pellet in early OVX’d mice does not result in the same reduction of this DC subset. Perhaps more interesting is the observation that a different subset of DCs (CD8a+) were not reduced in O4NP mice, and in fact trended toward in increase, suggesting that the “imprinting” that took place caused a shift in the DC developmental program, rather than simply being a survival signal. It is possible that levels of ERα were also altered at that time (although we do not have tissue from this early time point) since estrogen is known to increase ERα expression. One could speculate that when estrogen increases at the time of pubescence, it causes an increase in ERα expression that elicits a more proinflammatory environment that then informs DC development.

In summary, we investigated the effect of early OVX in WT lupus-prone animals and found the disease phenotype to be consistent with earlier reports in other lupus strains, except that autoantibody levels were actually increased in the (protected) early OVX’d mice, at both disease onset and late disease. Although there were not significant differences in survival, there was a trend toward survival benefit in the early OVX’d animals that were not estrogen repleted. These animals also had a significant reduction in CD11c+/CD11b+ DCs from spleen, which normally make up approximately 70% of murine spleen DC (45, 46). Conversely, tolerogenic CD11c+CD8a+ DCs were not reduced, but rather increased in O4NP mice, suggesting a mechanism for the protected phenotype. These data also suggest that the major impact of estrogen on DCs is early, specifically during the critical hormonal changes that take place at the time of puberty.

## Author Contributions

MC and GG directed the work, contributed to designing the study, and reviewed/interpreted the data. MC and JW conducted all studies with help from JE (breeding, genotyping, bleeding and urine collection), JS (IHC/flow cytometry), and EC (ELISAs). MC and JW prepared the manuscript and figures. GG contributed to manuscript revisions. All authors read and approved the final manuscript.

## Conflict of Interest Statement

The authors declare that the research was conducted in the absence of any commercial or financial relationships that could be construed as a potential conflict of interest.
